# Case report of a young stroke patient showing interim normalization of the MRI diffusion-weighted imaging lesion

**DOI:** 10.1186/s12880-015-0077-9

**Published:** 2015-08-25

**Authors:** Ann-Christin Ostwaldt, Tatiana Usnich, Christian H. Nolte, Kersten Villringer, Jochen B. Fiebach

**Affiliations:** Center for Stroke Research Berlin (CSB), Charité Universitätsmedizin Berlin, Hindenburgdamm 30, 12200 Berlin, Germany; Department of Neurology, Charité Universitätsmedizin Berlin, Hindenburgdamm 30, 12200 Berlin, Germany

**Keywords:** Magnetic resonance imaging, Acute ischemic stroke, Time course, Normalization

## Abstract

**Background:**

In acute ischemic stroke, diffusion weighted imaging (DWI) shows hyperintensities and is considered to indicate irreversibly damaged tissue. We present the case of a young stroke patient with unusual variability in the development of signal intensities within the same vessel territory.

**Case presentation:**

A 35-year-old patient presented with symptoms of global aphasia and hypesthesia of the left hand. MRI demonstrated a scattered lesion in the MCA territory. After rtPA therapy the patient received further MRI examination, three times on day 1, and once on day 2, 3, 5 and 43. The posterior part of the lesion showed the usual pattern with increasing DWI hyperintensity and decreased ADC, as well as delayed FLAIR positivity. However, the anterior part of the lesion, which was clearly visible in the first examination completely normalized on the first day and only reappeared on day 2. This was accompanied by a normalization of the ADC as well as an even further delayed FLAIR positivity.

**Conclusion:**

We showed that interim normalization of DWI and ADC in the acute phase can not only be found in rodent models of stroke, but also in humans. We propose that DWI lesion development might be more variable during the first 24 h after stroke than previously assumed.

## Background

Acute cerebral ischemia can be visualized with MRI diffusion weighted imaging (DWI) within minutes after its onset [1]. It has been suggested that in the majority of human stroke patients DWI positive cerebral ischemia indicate irreversible tissue damage leading to infarction [2]. However, lately reversal of parts of the DWI lesion has been demonstrated in association with early recanalization [3–5]. In animal models the occurrence of interim or sustained normalization of both DWI and apparent diffusion coefficient (ADC) seem to depend on the occlusion times [6, 7]. In contrast to DWI, lesions on fluid-attenuated inversion recovery (FLAIR) are usually seen several hours after stroke onset [8] in humans.

Here we present a case of partial interim normalization of DWI and ADC visualized with serial MRI in a young stroke patient.

## Case presentation

A 35 year old female patient was admitted with incomplete global aphasia and a fluctuating slight hypesthesia of the left hand. Her baseline National Institutes of Health Stroke Scale score (NIHSS) was 4 and arterial hypertension was the only vascular risk factor. The patient was repeatedly examined in the MRI 57 min as well as 3.3 h, 5.5 h and 7.6 h after symptom onset, and on the second, third, fifth and 43^rd^ day post-stroke. These data were obtained from the prospective 1000Plus Study (NCT00715533). The patient gave written informed consent. At baseline, the anterior M2 branch of the right middle cerebral artery (MCA) was occluded. DWI showed a scattered lesion affecting the anterior and posterior part of the right MCA territory and perfusion imaging showed a corresponding deficit. Intravenous rt-PA was administered directly after the first MRI scan. The follow-up examinations showed a patent MCA branch 5.5 h after symptom onset, complete normalization of the perfusion deficit on day 2 and no signs of hemorrhagic transformation. The patient’s symptoms had substantially improved, resulting in a NIHSS of 0 at discharge. Infarct size on FLAIR was 31.76 ml at day 5.

**Fig. 1:**
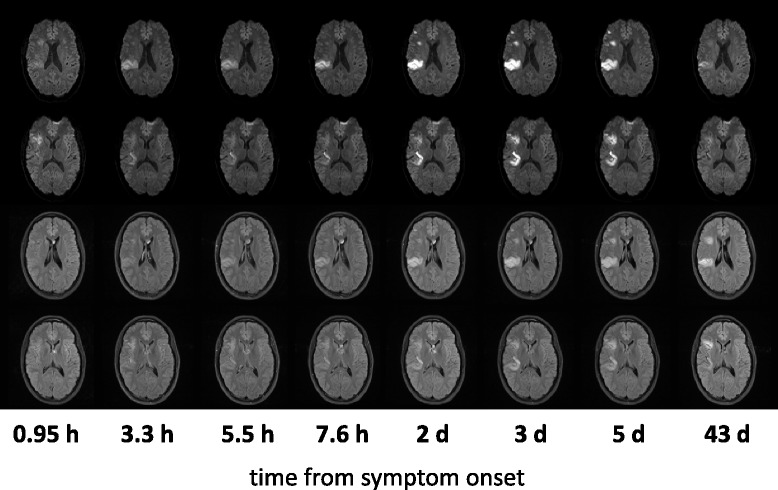
Development of the anterior and the posterior lesion on serial DWI and FLAIR images. The first and second rows show the diffusion-weighted images (DWI) on two slices depicting the lesion development over time from symptom onset. The third and fourth row shows the coregistered fluid-attenuated inversion recovery (FLAIR) images corresponding to the DWI slices

On the first examination 57 min after onset, the anterior part of the lesion was clearly hyperintense on DWI; however, on the second examination at 3.3 h it was not visible any more and did not reappear until day 2 (Fig. 1). For a more detailed analysis of this phenomenon, the image datasets for each time point were coregistered and regions-of-interest (ROIs) for both the anterior and the posterior part of the lesion were created on DWI day 5 images. Signal intensities (rSI) were determined relative to the contralateral side, as previously described [8].

**Fig. 2:**
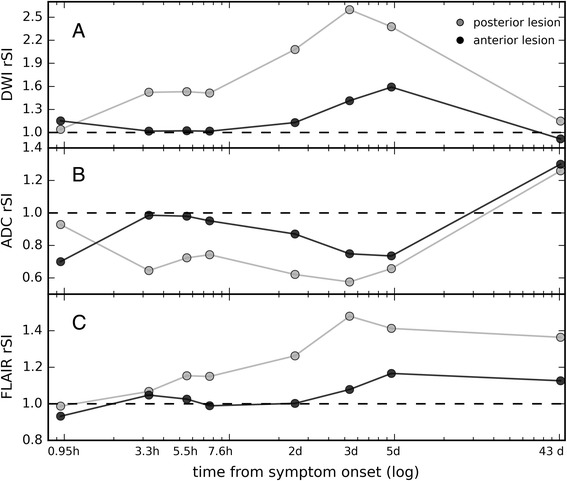
Development of relative signal intensity (rSI) for the anterior and the posterior lesion separately. **a** + **b**) Diffusion-weighted imaging (DWI) and apparent diffusion coefficient (ADC) rSI show the normalization of the anterior lesion and its reappearance at day 2. **c**) The fluid attenuated inversion recovery (FLAIR) rSI shows that the anterior part of the lesion is FLAIR positive much later than the posterior part

As can be seen in Fig. 2, DWI and ADC rSI values of the anterior lesion normalized for the time period between 3.3 h and 25 h after symptom onset (Fig. 2a-b). Also, the fluid-attenuated inversion recovery (FLAIR) rSI values of the anterior lesion were much lower than the values of the posterior lesion and only at day 3 post-stroke did the FLAIR rSI in the anterior lesion reach values substantially different from 1 (Fig. 2c).

## Conclusions

We demonstrated that the degree and time course of DWI hyperintensities can differ within one lesion in the first day after stroke. To the best of our knowledge, this is the first time that the exact time course of DWI reversal and reappearance was visualized using highly repetitive MRI examinations. Our findings resemble those in rodent models of stroke [6], where lesions normalized on the DWI and ADC after recanalization and reappeared at day 2 after stroke. In stroke patients, reversible DWI hyperintensities have been demonstrated for very small, mainly embolic lesions [9]. Also it has been shown that recanalization (either after intraarterial or intravenous thrombolysis) could evoke DWI lesion reversal in some patients, with parts of lesion showing later reappearance [3–5]. Therefore, we hypothesize, that a possible explanation for the interim normalization of a large part of the initial lesion in our case would be the fast recanalization and reperfusion of the tissue. It therefore seems that the baseline DWI lesion contains tissue with benign oligemia and only cell damage which could not be prevented will reveal itself on follow-up examinations. It might well be possible, that an interim normalization as observed in our case is more common in stroke patients. However, this will rarely be observed due to lack of MRI follow-ups in clinical routine.

We conclude that DWI lesion visibility might be more prone to fluctuation and variability during the first 24 h after stroke than previously assumed and baseline DWI may include tissue with benign oligemia. Therefore, during interpretation of DWI one has to keep in mind that an interim normalization can occur and which might delay lesion visualization.

## Abbreviations

MRI: Magnetic resonance imaging
DWI: Diffusion-weighted imaging
ADC: Apparent diffusion coefficient
NIHSS: National Institutes of health stroke scale
MCA: Middle cerebral artery
ROI: Region-of-interest
rSI: Relative signal intensity
FLAIR: Fluid-attenuated inversion recovery
